# Newly Described Copepod *Apocyclops cmfri* sp. nov. as a Potential Replacement for *Artemia* nauplii in the Larval Rearing of Vermiculated Spinefoot Rabbitfish (*Siganus vermiculatus*)

**DOI:** 10.1155/anu/7548728

**Published:** 2026-08-18

**Authors:** Suresh Babu Padinhate Purayil, Mini Kalapurakkal Gopalan, Sanal Ebeneezar, Anuraj Anirudhan, Tanveer Hussain, Mahendra Pal, Kiran Thomas Mathew, Sonali Suresh Mhaddolkar, Kalidas Chellappa, Suresh Vettath Raghavan, Shubhadeep Ghosh, Grinson George Padinjakara

**Affiliations:** ^1^ ICAR-Central Marine Fisheries Research Institute, Kochi 682018, Kerala, India, cmfri.org.in; ^2^ Karwar Regional Station of ICAR-Central Marine Fisheries Research Institute, Karwar 581302, Karnataka, India; ^3^ Indian Council of Agricultural Research, Division of Fisheries Science, Krishi Anusandhan Bhawan-II, New Delhi 110012, India, icar.org.in

**Keywords:** amino acid, copepods, fatty acid, live feeds, mouth gape, prey size suitability

## Abstract

This study evaluated the suitability of a new copepod, *Apocyclops cmfri*, as a live feed for larval rearing of *Siganus vermiculatus*. Total length (TL) and mouth opening (MO) of rabbitfish larvae increased exponentially with age (initial TL = 2.04 ± 0.096 mm; growth rate = 0.0657 day^−1^ ± 0.00166; *t*‐values for a and *b* = 21.13 and 39.69; *p*  < 0.001). Segmented regression analysis of MO–TL relationship identified a significant breakpoint at TL ≈ 8.96 mm (95% confidence interval), indicating a threshold for ingestion of *A. cmfri* adults and the gut content analysis confirmed the same. Feeding experiments from 22 day post hatch (dph), with larvae fed either *Artemia* nauplii alone at 5 or 1 individuals mL^−1^ or a combination of *A. cmfri* (4 individuals mL^−1^) and *Artemia* nauplii (1 individuals mL^−1^) revealed that at 50 dph, larvae fed *Artemia* nauplii alone at 5 individuals mL^−1^ achieved a length gain of 232.24% (survival 43.33%; metamorphosis rate 46.67%), which was not significantly different (*p*  > 0.05) from that fed the combination of *A. cmfri* adults and *Artemia* nauplii (222.37% length gain, 40.0% survival; 43.66% metamorphosis rate). Essential amino acids constituted 59.58% and 50.58% of the total amino acids in *A. cmfri* adults and unenriched *Artemia* nauplii respectively. In *A. cmfri* adults, EPA and DHA were 2.1 and 3.8% (in 1:1.81 ratio) whereas in unenriched *Artemia* nauplii, the content was minimal. These findings support the use of *A. cmfri* as a viable partial replacement to *Artemia* nauplii for sustainable marine hatchery practices.

## 1. Introduction

The vermiculated spinefoot rabbitfish, *Siganus vermiculatus*, is the fastest‐growing rabbitfish species, attaining a maximum weight of about 2 kg, and is tolerant to a wider range of salinity, temperature, and pH [1]. Their rapid growth, tender meat, strong consumer demand, high economic value, and herbivorous diet with low protein feed requirements make them an ideal species for mariculture. As a low‐trophic species, *Siganus* can be cultured using plant‐based feeds, reducing reliance on fishmeal and thereby improving the sustainability of aquaculture. Captive breeding and indoor larval rearing for the species have already been performed [2–4]. Earlier, Popper et al. [2] and Popper and Gundermann [3] relied on rotifers and wild copepods as feed for *S. vermiculatus* larvae. More recently, Anuraj et al. [4] added *Artemia* nauplii to this diet at a lower rate (<2 individuals per mL), which produced only marginal improvements in larval survival but introduced additional costs due to the need for *Artemia* and its DHA enrichment.

Live feed management plays a major role in the successful development of hatchery technology for marine finfishes. Lipids, which provide about two‐thirds more energy than proteins or carbohydrates, serve as the primary metabolic fuel, supporting the rapid growth and development of larvae. Three long‐chain polyunsaturated fatty acids, docosahexaenoic acid, eicosapentaenoic acid, and arachidonic acid, are considered crucial for larval development [5]. Even though *Artemia* and rotifers are preferred as the most suitable live feed for larval rearing of marine finfishes due to their ease of maintenance and wide availability, they possess low levels of essential fatty acids, particularly docosahexaenoic acid and eicosapentaenoic acid, that are not adequate for larval stages and therefore must be enriched prior to their use as prey [6]. Contrastingly, copepods, the natural prey in the wild, are rich in phospholipids and essential fatty acids [7]. Also, as over 50% of the larval composition is protein, supplying dietary protein of the right quality and quantity is paramount for optimizing growth [8]. Growth, feed conversions, immune functions, and survival are maximized by providing an ideal composition of essential fatty acids and amino acids. Conversely, their imbalances in the diet lead to reduced efficiency, ultimately limiting growth performance and survival.

Copepods are ubiquitous planktonic organisms inhabiting diverse aquatic environments that are recognized as beneficial live feed organisms for fish larvae [9, 10]. The mass production of several copepods for marine finfish larval rearing has been developed and standardized [11–13]. Copepod nauplii are a rich source of fatty acids [13–15] and also contain higher levels of dietary taurine (a nutritional factor influencing organ development) [16, 17]. Due to their smaller size and superior nutritional profile than other live feeds, copepods are considered ideal for larval rearing in various fish species [18]. Members of the cyclopoid genus *Apocyclops* have been identified as potential live feed organisms for commercial aquaculture. Prominent candidates such as *Apocyclops royi* [19], *Apocyclops dengizicus* [20], and *Apocyclops panamensis* [21] have been developed for feeding larvae of commercially important fish and shrimp. A new species, *Apocyclops cmfri* sp. nov. (Cyclopoda: Cyclopoida: Cyclopidae) was isolated from Indian waters by Loka et al. [22], and the culture prospects of this species have been evaluated and standardized [13, 23], establishing it as a promising live feed option for larval rearing of fish and shrimps [13, 23].

Key factors for the selection of appropriate live feed include the size, motility, and nutritional profile of the feed, as well as its digestibility and availability [24]. It is ideal to find out the acceptability of a new live feed organism prior to its introduction into the larval rearing protocols of any marine finfish. Prey size and mouth opening (MO) of the larvae play a crucial role in determining the acceptability of the prey to the fish larvae [25, 26]. The mouth gape varies in fish larvae during development. The mouth gape–larval length relationship has been effectively employed as a predictive model in commercial fish larvae [27, 28].

In the present study, the suitability of the adult copepods of the species *A. cmfri* as a live feed for the larvae of the vermiculated spinefoot rabbitfish, *S. vermiculatus*, was evaluated. A predictive model for finding out the relationship between MO and total larval length was also proposed to find out the minimum length required for the larvae to ingest the adult *A. cmfri* copepod since it is a larger prey ideal to replace *Artemia* nauplii at the later stage of rearing. The results were validated by conducting feeding trials on the larvae using various life stages of this new copepod species.

## 2. Materials and Methods

### 2.1. Ethical Statement

The work presented in this manuscript adheres to the ethical guidelines for conducting experiments on food fishes in India, as per the regulations set forth by the Committee for the Purpose of Control and Supervision of Experiments on Animals (CPCSEA), India. The research work was carried out at the Marine Hatchery Complex of the Karwar Regional Station of ICAR – Central Marine Fisheries Research Institute, Karwar, Karnataka, India.

### 2.2. Live Feed

#### 2.2.1. Phytoplankton

Microalgal species used in this study were obtained from the algal culture unit of ICAR – Central Marine Fisheries Research Institute (ICAR‐CMFRI), Research Centre Karwar, India. Three species were maintained: the Tahitian strain of *Isochrysis galbana*, *Nannochloropsis oculata*, and the diatom *Chaetoceros calcitrans*. Cultures were grown in 20 L polycarbonate carboys containing filtered, UV‐irradiated seawater at 30 ppt salinity under thermally regulated conditions (24 ± 1°C). *N*. *oculata* was cultured in f/2 medium [29], while *I*. *galbana* and *C*. *calcitrans* were maintained in Walne medium [30] with continuous aeration. Illumination was provided for 24 h at 3000 lx, measured using a lux meter (Lx‐101A, HTC, India). Cell densities were determined using a hemocytometer under a trinocular microscope (LM52180, Lynx Lawrence & Mayo, India). Algal cells were harvested during the exponential growth phase for experimental use.

#### 2.2.2. Zooplankton

The rotifer, *Brachionus rotundiformis*, maintained at Research Centre Karwar, was cultured in 1000 L fiber‐reinforced plastic (FRP) tanks and fed with *N*. *oculata* and *I*. *galbana*. Harvesting was carried out by sieving through a 40 µm mesh, followed by rinsing in seawater. The collected rotifers were transferred to 100 L tubs, where population density was determined using a zooplankton counting chamber under a microscope by averaging the counts from three 1 mL subsamples. For enrichment, rotifers were maintained in 100 L tubs with strong aeration and supplemented with Algamac 3050 flake (Aquafauna Bio‐Marine) for 8 h prior to larval feeding to enhance their fatty acid composition.

The stock culture of calanoid copepod, *Parvocalanus crassirostris*, was obtained from Vizhinjam Regional Centre of ICAR‐CMFRI, Kerala, India. A 20 L stock culture with an initial density of 2000 individuals L^−1^ was transferred into 1000 L tanks for mass production. Within 14–16 days, the population reached its peak density. Thereafter, daily harvests were conducted by filtering through a 35 μm sieve, and the harvest consisted of a combination of eggs, nauplii, and adults. Eggs and nauplii were further separated from copepodites and adults by sieving through a 70 μm mesh [31].

The copepod, *A. cmfri*, was isolated and maintained at the ICAR‐CMFRI Research Centre, Karwar. Stock cultures, initially maintained in 20 L plastic tubs at a density of 1 individuals mL^−1^, were upscaled by transferring into 1000 L FRP tanks filled with sterile filtered seawater (32 ppt) and maintained at a temperature of 28 ± 1°C, natural light regime, and gentle aeration. The culture was fed *C. calcitrans* (7 × 10^4^ cells mL^−1^) once daily in the morning and maintained for 30 days with minimal water exchange (5%–10%) and regular cleaning. The whole culture was harvested using a 60 μm mesh once in every 15–20 days, upon attaining a copepod density of 2–3 individuals mL^−1^, which was sufficient for the present feeding regime, and a fresh culture was initiated using a portion of eggs and nauplii produced from this culture. The eggs, nauplii, copepodites, and adults retained on a 60 μm mesh were segregated using a 170 μm sieve to separate bigger copepodites and adults from smaller eggs and nauplii [13].

*Artemia salina* nauplii were produced from commercial *Artemia* cysts (OSI, USA) using standard protocols described by Campton and Busack [32]. The umbrella stage was harvested after 12 h of incubation and separated out following Nhu et al. [33]. *Artemia* nauplii were enriched with DHA Selco (Inve, Belgium) following the manufacturer’s protocol and used for routine larval rearing practices only.

### 2.3. Determining the Suitability of *A. cmfri* Adult as Live Feed for Rabbitfish Larvae

#### 2.3.1. Morphological Measurements of *A. cmfri* Adults and *Artemia* nauplii

*A. cmfri* adults, filtered from their rearing tanks by a 300 µm mesh, were studied morphologically under the microscope. The total length (TL; from tip of the rostrum to the end of the urosome) and maximum width (W; widest part of the cephalo–thorax) were measured from 30 individuals using a compound research microscope (Zeiss Axio Scope A1) featuring a digital camera (Jenoptik ProgRes C3) at 10× magnification. Similarly, TL and W of *Artemia* nauplii were also recorded from 30 individuals each.

#### 2.3.2. Morphological Measurement of Fish Larvae

The breeding and larval rearing protocols for S. vermiculatus followed by Anuraj et al. [4] were adopted. Fertilized eggs attached to the tiles were incubated in 1000 L FRP tanks (larval rearing tanks) containing 0.8 t of dechlorinated seawater (32 ppt) with gentle aeration. A green‐water system using *N. salina* and *I. galbana* (2 × 10^6^ cells mL^−1^) was adopted for larval rearing at 28–30°C. Larvae were fed copepod, *P. crassirostris* (3 nauplii per mL), from 1 to 10 day post hatch (dph) and co‐fed with rotifer, *B. rotundiformis*, from 5 to 35 dph (5–10 individuals mL^−1^), and enriched *Artemia* nauplii from 20 to 35 dph (2–5 individuals mL^−1^). From the larval rearing tank, ~20 live larval samples were collected at 3 day intervals from 0 to 36 dph and analyzed using a compound research microscope (Zeiss Axio Scope A1) featuring a digital camera (Jenoptik ProgRes C3) (up to 10 dph) and a stereo zoom microscope (ZEISS Stemi 508; China) with an inbuilt digital camera (up to 36 dph). The larvae were anesthetized using clove oil (0.1 ppm) in ice‐cold water before observation. The TL of larvae was measured from snout to tail tip. The MO of larvae was quantified as the maximum distance between their lower and upper jaws in the opened condition, observed under a cover slip on a glass slide as per Suresh Babu et al. [34].

#### 2.3.3. MO and TL Relationship in Larvae

The dataset consisted of MO (mm) as a dependent variable and TL (mm) of fish larvae as an independent variable, collected from individual specimens (*n* = 482) at various dph. To linearize the relationship and stabilize variance, both length and mouth size were log‐transformed and used for all regression modeling. First, a simple linear regression model was fitted by plotting as per Krebs and Turingan [35]. Then, a segmented regression model was fitted using the segmented package with an initial breakpoint guess. The model performance was assessed by comparing the segmented model to the initial linear model.

#### 2.3.4. Prediction of Optimum Fish Larval Size for Feeding *A. cmfri* Adults

The regression equation was used as a predictive model to predict the minimum larval length required to consume live feed organisms. The predicted minimum length was calculated by setting the MO as twice the average width of the live feed organism, as per Jackson and Lenz [36]. The following equations were used for the same purpose.

For logx<2.192;logy=a+blogx,

For logx≥2.1922.192;logy=a+b1×+b2×logx−2.192,

where *x* is MO (mm), *y* is TL, *b* is the slope, and *a* is the intercept.

To find the larval age (dph) corresponding to the TL, a predictive model of larval growth over time was designed from the average length data of rabbitfish larvae on alternate days from 4 to 30 dph. To describe the pattern of larval growth over time, a nonlinear regression model was applied, assuming an exponential form.

Length=aeb+Day,

where, length is the observed TL in mm, day is the age of the larva in dph, *a* is the estimated initial length at Day 0, and *b* is the exponential growth rate.

#### 2.3.5. Acceptability of *A. cmfri* Adult as Feed by Larvae

To confirm the acceptability of *A. cmfri* adults by fish larvae, an initial feeding trial was conducted, and the gut contents were evaluated. Three rectangular plastic tubs (44 cm × 32 cm × 18 cm), each containing 10 L of 32 ppt seawater with aeration, were stocked with 50 numbers of 30 dph fish larvae (5 larvae L^−1^) and fed adult *A. cmfri* at 3 individuals mL^−1^. This feeding regimen was sustained for 3 days by replenishing the adult copepods. At the end of the experimental feeding trial, microscopic observation of gut content from 5 larval samples from each tub was carried out following the methodology delineated by Sarvi et al. [37] to assess their feed intake.

### 2.4. Evaluation of *A. cmfri* Adult as an Alternate Live Feed to *Artemia* nauplii in Larval Rearing of Rabbitfish

*S. vermiculatus* larvae were collected from the marine finfish hatchery complex of the Research Centre at Karwar on 22 dph from a larval rearing tank. The feeding trial was initiated at 22 dph, when larvae had attained the minimum mouth size necessary to ingest both adult *A*. *cmfri*. The experiments were conducted in triplicate. For this, 9 rectangular plastic tubs (60 cm × 42 cm × 24 cm), each containing 30 L of 32 ppt seawater, were stocked with 150 fish larvae (5 L^−1^) with sufficient aeration and light (1000 Lx). Uniform‐sized larvae were selected manually by visual observation. Rabbitfish larval stocking density and the green‐water feeding regime followed protocols from our earlier study [4]. With no optimized mid‐larval stocking rate, the present density was determined using biomass observations reported previously. In the first three tubs, larvae were exclusively provided with enriched *Artemia* nauplii at a density of 5 individuals mL^−1^. In the second set of three tubs, adult *A. cmfri* was offered to the larvae at 4 individuals mL^−1^ along with enriched *Artemia* nauplii at 1 individual mL^−1^. In the third set of three tubs, building on our previous study [4,], enriched *Artemia* nauplii were provided at 1 individual mL^−1^, and this served as the control baseline. Since no standardized feeding protocol exists for this species, the present control was adopted accordingly. A higher *Artemia* density (control + 4 nauplii mL^−1^) was tested, following global research that reported improved outcomes in tropical fish species. Additionally, this combination (control + 4 copepod mL^−1^) was evaluated to assess the suitability of adult *A. cmfri* replacement. Also, the present control served to determine if the larval survival noted in the combined feeding with adult *A. cmfri* and *Artemia* nauplii was attributable exclusively to the latter. This feeding regimen was sustained till 50 dph. Daily counts of *Artemia* nauplii and *A*. *cmfri* adults were conducted in triplicate from the entire seawater volume of each tank during early morning hours prior to siphoning and water exchange. These subsamples collected in the morning from each tank, prior to replenishment, provided residual counts to estimate larval consumption patterns. Enumeration was performed using a plankton counting slide under light microscopy. To maintain the required densities, freshly filtered live feeds were supplemented after each count. Residual numbers of live feeds in the rearing water were recorded, and daily reductions were used to indirectly estimate larval ingestion rates across treatments. The average larval survival rates were evaluated at 30, 40, and 50 dph. The TL of the fish larvae was measured at 22 and 50 dph from each tank by sampling 30% of the initial stocking numbers.

The average fish larval survival rate was calculated as follows:

Larval survival (%)=(Final number of larvae)(Initial number of larvae)×100.

The percentage length increment of fish larvae was calculated as follows:

Percentage length increment (mm)=Final length−Initial lengthInitial length×100.

The percentage of metamorphosed fish larvae was expressed as follows:

Metamorphosed larvae (%)=Number of metamorphosed larvae×100Initial number of larvae.

### 2.5. Nutritional Profile of *A. cmfri* and Unenriched *Artemia* nauplii

#### 2.5.1. Fatty Acid

The *A. cmfri* adults and unenriched *Artemia* nauplii were filtered and collected aseptically from two of their rearing tanks. The biomass for each was subjected to saponification using 0.5 N sodium hydroxide in methanol, followed by methylation with 14% boron trifluoride (BF_3_) in methanol to produce fatty acid methyl esters (FAMEs). The resulting FAMEs were analyzed using gas chromatography on a Perkin Elmer Autosystem XL, equipped with an Elite 225 capillary column (30 m × 0.25 mm × 0.25 μm) and a flame ionization detector (FID). Nitrogen was used as the carrier gas at a flow rate of 0.5 mL/min. The injector temperature was set at 265°C. The oven temperature was initially held at 110°C for 5 min and then increased to 240°C at a rate of 2.7°C/min. After reaching 240°C, the temperature was maintained for 3 min, followed by a further increase to 280°C, which was held for 5 min. Identification of individual fatty acid components was achieved by comparing the retention times with those of a standard FAME mixture (Supelco 37 Component FAME Mix, 100 mg neat, Sigma–Aldrich, Catalog Number 18919‐1AMP). The results were reported as weight percentages.

#### 2.5.2. Amino Acid

Homogenized biomass of *A. cmfri* adults (two samples) and unenriched *Artemia* nauplii (two samples) was taken in a sealing test tube. To this, 10 mL of 6N hydrochloric acid was added, and the tube was then sealed securely. The sealed tube was placed in a hot air oven and incubated at 110°C for 24 h to facilitate hydrolysis. After hydrolysis, the sample was filtered through a Whatman filter paper into a flat‐bottom flask. The filtrate was then evaporated to dryness using a rotary evaporator set at 80°C. Following this, 20 mL of distilled water was added to the residue in the flask, and the mixture was again concentrated using a rotary evaporator. This process of adding distilled water and concentrating was repeated one more time to ensure the removal of the residual acid. Finally, the samples were concentrated to dryness, and 5 mL of 0.05 N hydrochloric acid was added to the flat‐bottom flask. The prepared solution was transferred into a 5 mL vial and stored in a refrigerator until further analysis. Amino acids were analyzed following acid hydrolysis using high‐pressure liquid chromatography (HPLC; Waters, USA).

### 2.6. Statistical Analyses

Regression models between MO and TL in rabbitfish larvae were compared using analysis of variance (ANOVA). The statistical significance of the breakpoint and its associated confidence interval were computed. The final model’s fit was evaluated using the adjusted R‐squared and residual standard errors. All analyses were performed using R version (4.4.2) [38].

A regression model for predicting the minimum larval length required to consume the copepod adults was fitted using the minpack.lm package in R [39]. Model performance was evaluated based on the residual standard error. Confidence intervals for predicted lengths were derived using nonparametric bootstrapping (1000 replicates) with the bootstrap package. The 2.5^th^ and 97.5^th^ percentiles of the bootstrapped predictions were used to construct 95% confidence bands around the fitted curve. Residual diagnostics included plots of residuals versus fitted values and Q–Q plots to assess normality and homoscedasticity. All analyses were conducted using R version 4.4.1, and the package bootstrap was used for bootstrapping [40].

The percentages of larval survival, length increment, and metamorphosed larvae following feeding copepod adults are presented as mean ± standard error. The mean values were compared using a one‐way ANOVA for larval survival, *t*‐test for length increment and metamorphosed larvae to determine significant differences (*p* < 0.05).

The content of each amino acid and fatty acid was compared using *t*‐test among treatment groups. Statistical analysis was performed using SPSS Version 16.0.

## 3. Results and Discussion

Live feeds are essential for the successful larval rearing of various rabbitfish species (family *Siganidae*), including *Siganus guttatus*, *S. javus*, and *S. fuscescens*. These species exhibit high mortality during early larval stages, primarily due to underdeveloped digestive systems and poor initial feeding responses [41, 42]. Live feeds not only stimulate feeding behavior through their natural movement but also provide superior digestibility and nutritional value compared to inert feeds. Rotifers are commonly used as the first feed, typically offered from 2 to 3 dph when larvae begin exogenous feeding. As the larvae grow, *Artemia* nauplii is introduced, providing a larger prey item with higher protein content. Like rotifers, *Artemia* must also be enriched with essential fatty acids to prevent nutritional deficiencies that impair larval development [43]. Copepods, with higher natural levels of DHA when included in larval diets, have been found to significantly improve larval growth, survival, and skeletal development [44]. However, due to the challenges associated with their mass culture, copepods are less commonly used.

### 3.1. Suitability of *A. cmfri* Adult as Live Feed for Rabbitfish Larvae

**Figure 1:**
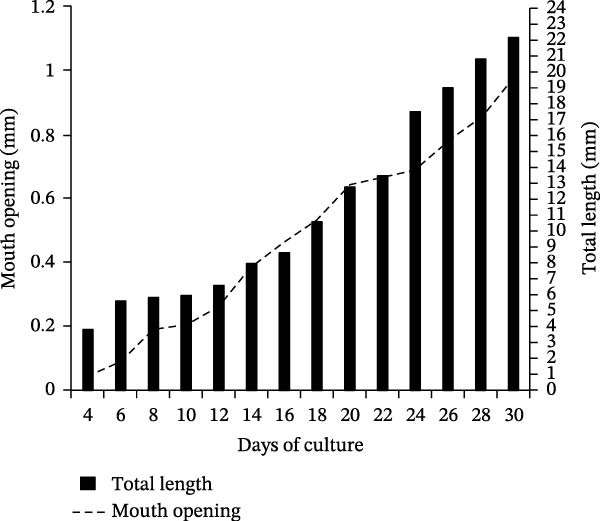
Measurement of total length and mouth opening of rabbitfish larvae at various larval stages (dph).

**Figure 2:**
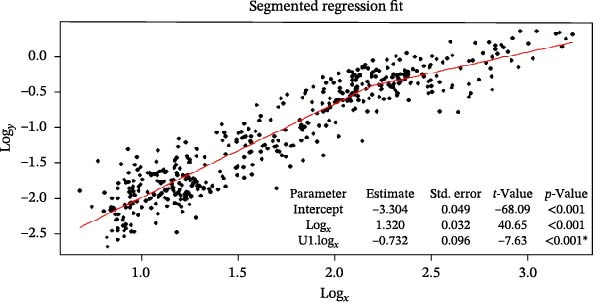
Mouth opening and total length relationship in rabbitfish larvae by segmented regression analysis.

The TL and W of the live feed organisms, *A. cmfri* and *Artemia* nauplii, were recorded and are presented in Table 1A. Measurements of TL and MO of rabbitfish larvae at various larval stages (dph) are plotted in Figure 1. The TL and MO increased gradually as the larval development proceeded. The segmented regression analysis (Figure 2) identified a statistically significant breakpoint in the log‐transformed predictor variable at log_*x*_ = 2.192, with a 95% confidence interval of (2.064, 2.320). When back‐transformed to the original scale, this corresponded to a breakpoint at *x* ≈ 8.96 mm, with a 95% confidence interval of (7.88 and 10.18 mm). This indicated a distinct change in the relationship between the variables occurring near this value of length. Before the breakpoint, the slope was ~1.32, indicating a strong positive association. Following the breakpoint, the slope decreased to ~0.59, reflecting a notable reduction in the rate of change. This shift suggested a structural change in the relationship between TL and MO at the estimated breakpoint. The segmented regression model significantly outperformed the simple linear model, effectively capturing a structural shift in the TL–MO relationship that may reflect a biological threshold.

**Table 1:** Morphological measurements and predicted fish larval size optimum for feeding various live feeds.

A. Morphological measurements					
	Total length (mm)		Body width (mm)		
*Apocyclops cmfri* adult	1.14 ± 0.012		0.38 ± 0.008		
*Artemia* nauplii	0.48 ± 0.006		0.21 ± 0.008		

**B. Predicted fish larval size optimum for feeding various live feeds**					

	Mouth opening (*Y*) (width of zooplankton × 2) in mm	Log_*y*_	Log_*x*_	*X* (length in mm)	Corresponding larval age (dph)
*Apocyclops cmfri* adult	0.38	−0.42022	2.183673	8.878858	22
*Artemia* nauplii	0.21	−0.67778	1.988537	7.304836	16

**Figure 3:**
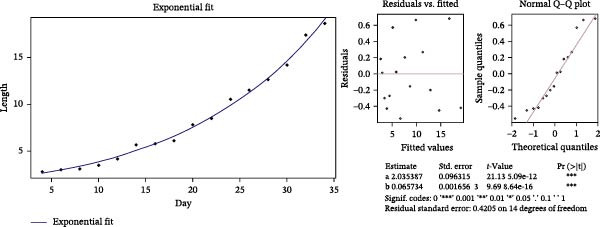
Exponential relationship of total length of *S. vermiculatus* larvae and age of larvae (day) derived from the morphometric analysis.

An exponential growth model was fitted to the day‐wise length data of the larva (Figure 3). The model provided an excellent fit to the observed data, with both estimated parameters being highly significant. The initial length at Day 0 (*a*) was estimated at 2.04 mm (±0.096 mm SE), while the exponential growth rate (*b*) was 0.0657 day^−1^ (±0.00166 SE). The *t*‐values for parameters *a* and *b* were 21.13 and 39.69, respectively, with *p*‐values well below 0.001, indicating strong statistical significance. The residual standard error was 0.4205 mm, reflecting a close alignment between the observed and predicted lengths across the larval development period. The results confirmed that larval growth over time during the observed phase followed a clear exponential pattern.

From the segmented regression analysis and the exponential plot, the minimum length (*X*) of rabbitfish larvae required for consuming *A. cmfri* adults was evaluated (Table 1B). According to Jackson and Lenz [36], the minimum MO of the fish larvae required for ingesting adult copepods is double the width of the copepod. From the exponential plot, it is predicted that *A. cmfri* adults are expected to be prey for *S. vermiculatus* larvae of TL 8.9 mm at an age of 22 dph and *Artemia* nauplii to be a prey at TL 7.3 mm at an age of 16 dph and above.

The choice of live feed for larvae depends on their developmental stage and size, as demonstrated in several studies [45, 46]. Prey selection is a critical factor for the successful larval rearing of fish. Key factors influencing prey selection in fish larvae include the mouth gape of the larvae [47, 48], prey abundance [45], and environmental factors like light intensity [49]. MO is predominantly considered for determining prey selection in fish larvae [27]. Optimally, the prey size should be less than half the estimated larval mouth gape [50]. The present analysis revealed that only at 22 dph, the fish larvae attained the minimum MO required for ingesting adult *A. cmfri*, indicating the larvae’s inability to prey on copepod adults before this stage. An earlier study on turbot larvae revealed a logarithmic relationship between mouth size (width and height) and standard length [25].

**Figure 4:**
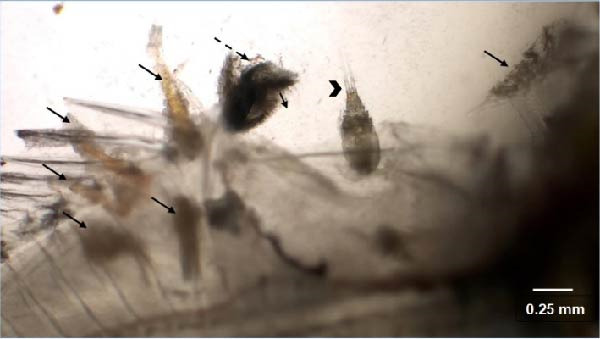
Gut content of 33 dph *S. vermiculatus* larvae indicating the presence of remnants of *A. cmfri* adult copepods (black arrow) and *A. cmfri* nauplii (dotted arrow). A live adult copepod is also visible in the background (arrowhead).

*Apocyclops* is one of the most widely used copepod genera in aquaculture. The gut content analysis of 33 dph larvae revealed the presence of adult *A. cmfri* (Figure 4). The potential for developing this copepod as a live feed for larval rearing of marine finfish, especially during the middle to later larval rearing phases before and during metamorphosis was evaluate. Limited data exist on how *Artemia* withstands environmental fluctuations, mostly high temperature and low salinity [51]. In extreme environments, the high PUFA content in these enrichments is reported to produce harmful compounds [52]. Therefore, the possibility of replacing *Atemia* nauplii during larval rearing and eliminating the risks and expenses associated with their enrichment by using the adults of *A. cmfri* is expected to be a boon for marine hatchery enterprises in tropical countries.

### 3.2. Evaluation of *A. cmfri* Adult as an Alternate live Feed to *Artemia* nauplii in Larval Rearing of Rabbitfish

**Figure 5:**
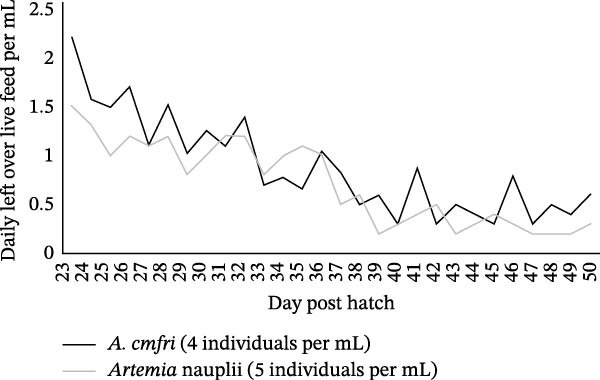
Daily residual counts of adult *A. cmfri* and *Artemia* nauplii maintained at 4 and 5 individuals mL^-1^ during the feeding experiment.

It was observed that in all the replicates, where *Artemia* nauplii was provided at 1 individual mL^−1^, it was totally consumed. Daily residual counts of adult *A. cmfri* and *Artemia* nauplii maintained at 4 and 5 individuals mL^−1^ during the feeding experiment are provided in Figure 5. With an increase in the days of rearing, there was a gradual decrease in the residual counts of both adult *A. cmfri* and *Artemia* nauplii, possibly indicating greater feed consumption and feeding frequency. During the first and last few days, the residual counts were higher for adults of *A. cmfri* in comparison to those of *Artemia* nauplii. However, for the majority of the rearing days, no major difference in counts was observed. Statistical comparisons were not performed due to variation in feeding rates; however, from Figure 5, it is apparent that ingestion rates were mostly similar.

**Figure 6:**
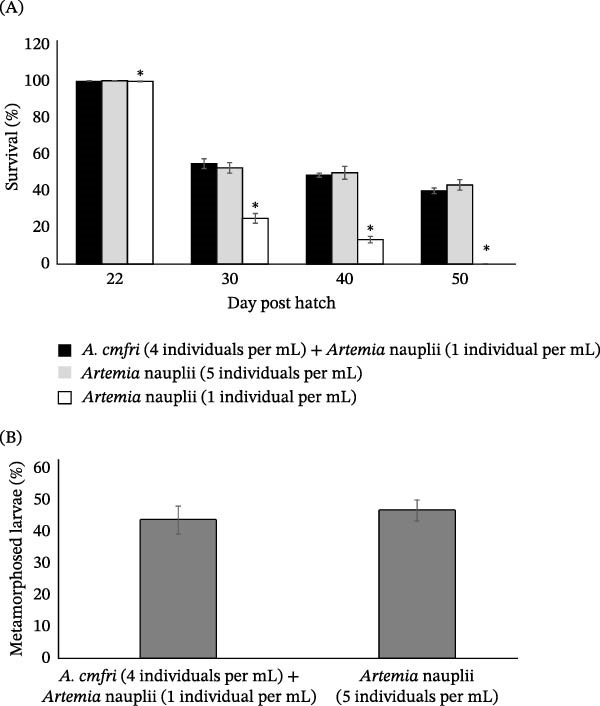
Histogram indicating larval survival (%) (A) and percentage of metamorphosed larvae (B) observed during the feeding experiment using *A. cmfri* adult and *Artemia* nauplii. Bars with  ^∗^ differ significantly from the other treatments on a particular day post hatch.

Larval survival on 30, 40, and 50 dph upon feeding *A. cmfri* adult and *Artemia* nauplii is given in Figure 6A. When fed *Artemia* nauplii at a lower rate of 1 individual mL^−1^, survival was significantly lower (*p* < 0.05) at 30 and 40 dph, and 100% mortality was observed by 50 dph. No larvae were found to metamorphose in this feeding protocol. Larval survival did not differ significantly (*p* > 0.05) between the larvae fed 5 individual mL^−1^ *Artemia* nauplii and “4 individual mL^−1^ *A. cmfri* + 1 individual mL^−1^ *Artemia* nauplii,” with marginally higher survival observed in the later on 30 dph, and in the former on 40 and 50 dph. The percentages of metamorphosed larvae also did not vary significantly (*p*  > 0.05) between these two feeding regimes (Figure 6B), with slightly higher values recorded in the feeding with *Artemia* nauplii at 5 individual mL^−1^ (46.67% vis‐à‐vis 43.66%). Therefore, it is concluded that partial substitution of four *Artemia* nauplii per mL with an equal number of *A. cmfri* adults in the feeding procedure of rabbitfish larvae did not produce perceptible changes in larval survival and metamorphosis. However, as the survival and metamorphosis percentages in larvae fed solely on *Artemia* nauplii were marginal; hence, complete replacement of *Artemia* nauplii with *A. cmfri* adults was not further attempted.

The growth performance of rabbitfish larvae fed solely on *Artemia* nauplii (5 individual mL^−1^) exhibited a higher percentage increment in TL (232.24 ± 8.29%) compared to those reared on the mixed diet of *Artemia* nauplii and *A. cmfri* adult (222.37 ± 12.47%) (Table 2). However, the differences in TL between the two feeding practices at 50 dph were not statistically significant (*p*  > 0.1). This indicated that while both feeding regimes supported larval growth, *Artemia*‐based feeding alone resulted in a comparatively higher length gain, although the effect was not of statistical significance (*p*  > 0.1).

**Table 2:** Growth performance of *Siganus vermiculatus* larvae fed with various live feeds.

Treatment	Total length (cm)		
	22 dph	50 dph	Percentage increment length
*Artemia* (1 individual mL^-1^) + *A. cmfri* (4 individuals mL^-1^)	1.52 ± 0.00	4.90 ± 0.19	222.37 ± 12.47
*Artemia* (5 individuals mL^-1)^	1.52 ± 0.00	5.05 ± 0.13	232.24 ± 8.29
*p*‐Value	–	>0.1	>0.1

Various stages of copepods are employed as live prey for fish larvae, depending on the larval size [53]. Anuraj et al. [4] standardized the feeding protocol for rabbitfish larvae, recommending the copepod, *P. crassirostris* nauplii (0–10 dph), rotifer, *B. plicatilis* (10–38 dph), and *Artemia* nauplii (18–38 dph). Replacing *Artemia*, an expensive live feed in hatcheries, has been previously tried in marine fish [54–56]. In this study, an attempt was made to partially substitute *Artemia* nauplii with *A. cmfri* adults as a live feed from 22 dph of larval development due to their size similarity. The larval length increment, larval survival, and larval metamorphosis were not impacted by this effort, as evident from the comparable results. Therefore, the present findings substantiate the potential of this cyclopoid species in marine larviculture. The ability to feed on almost all species of microalgae, coupled with its tolerance to a wider range of temperatures (15–32°C) and salinities (20–40 ppt), makes it an ideal live feed for the early life stages of tropical estuarine and coastal fish [23].

### 3.3. Nutritional Profile of *A. cmfri* and Unenriched *Artemia* nauplii

The efficiency of live prey depends on the provision of sufficient amounts of amino acids and unsaturated fatty acids for the growth and development of larvae. The amino acid and fatty acid compositions of *A. cmfri* adults and unenriched *Artemia* nauplii are presented in Table 3. The essential amino acids (EAAs) constituted 59.58% and 50.58% of the total amino acid content in *A. cmfri* adults and unenriched *Artemia* nauplii, respectively. Arginine, threonine, tyrosine, valine, isoleucine, leucine, and phenylalanine were significantly (*p*  < 0.05) higher in *A. cmfri* adults, while histidine was significantly higher (*p*  < 0.05) in unenriched *Artemia* nauplii. Temperate calanoid copepods are reported to carry substantial protein stores (33%–54% of dry weight as protein‐bound amino acids), with a stable EAA share exceeding 40% within the protein pool [14]. The EAA content of this tropical copepod, *A. cmfri* was notably higher than that earlier reported from temperate water species (∼50%) [8]. Together, the EAAs surpassed the limiting values typically reported in other commonly used live feeds, thereby reducing the risks of amino acid deficiencies that impair larval growth and protein accretion [57]. On amino acids, copepods are typically adequate but variable [58], and present findings indicate a favourable amino acid and fatty acid profile of *A. cmfri* compared to *Artemia* nauplii.

**Table 3:** Amino acid and fatty acid composition of *Apocyclops cmfri* and unenriched *Artemia* nauplii.

Sr. number	Amino acid	*Apocyclops cmfri* (%)	*Artemia* nauplii (%)	Fatty acid	*Apocyclops cmfri* (%)	*Artemia* nauplii (%)
1	His	1.83 ± 0.12^a^	4.43 ± 0.25^b^	C 12:0 – Lauric acid	2.3 ± 0.03	—
2	Arg	8.52 ± 0.35^a^	6.42 ± 0.21^b^	C 14:0 – Myristic acid	6.0 ± 0.15^a^	1.2 ± 0.12^b^
3	Thr	5.63 ± 0.43^a^	3.65 ± 0.09^b^	C 16:0 – Palmitic acid	19.7 ± 0.12^a^	15.6 ± 0.08^b^
4	Lys	4.63 ± 0.27	5.62 ± 0.21	C 18:0 – Stearic acid	10.0 ± 0.06^a^	7.5 ± 0.08^b^
5	Tyr	6.94 ± 0.31^a^	4.96 ± 0.16^b^	C 17:0 – Heptadecanoic acid	—	2.4 ± 0.07
6	Met	4.63 ± 0.19	4.52 ± 0.13	C 23:0 – Tricosanoic acid	1.2 ± 0.04	—
7	Val	6.1 ± 0.26^a^	4.54 ± 0.18^b^	C 18:1 – Oleic acid	24.7 ± 0.18^a^	26.1 ± 0.11^b^
8	Ile	4.91 ± 0.29^a^	3.16 ± 0.20^b^	C 16:1 – Palmitoleic acid	3.9 ± 0.80	4.6 ± 0.12
9	Leu	8.64 ± 0.29^a^	6.72 ± 0.29^b^	C 14:1 – Myristoleic acid	—	1.7 ± 0.05
10	Phe	7.75 ± 0.38^a^	6.56 ± 0.18^b^	C 17:1 – Heptadecenoic acid	—	2.3 ± 0.06
11	Ser	5.78 ± 0.06^a^	7.16 ± 0.26^b^	C 20:1 – Eicosenoic acid	2.5 ± 0.18	2.1 ± 0.03
12	Gly	9.84 ± 0.43	10.8 ± 0.34	Other MUFA	0.5 ± 0.01	1.1 ± 0.03
13	Asp	4.76 ± 0.09^a^	8.28 ± 0.19^b^	C 18:3 – Linolenic acid	6.2 ± 0.06^a^	23.3 ± 0.03^b^
14	Glu	4.35 ± 0.22^a^	9.88 ± 0.18^b^	C 18:3 – Gamma linolenic acid	1.3 ± 0.04^a^	0.4 ± 0.09^b^
15	Ala	8.35 ± 0.36^a^	6.02 ± 0.07^b^	C 20:5 – Eicosapentanoic acid	2.1 ± 0.12^a^	0.4 ± 0.07^b^
16	Pro	6.05 ± 0.25	7.28 ± 0.15	C 22:6 – Docosahexanoic acid	3.8 ± 0.07^a^	0.1 ± 0.03^b^
17	Cys	1.3 ± 0.17	—	C 18:2 – Linoleic acid	14.3 ± 0.06^a^	9.8 ± 0.07^b^
EAA as % of total AA		59.58	50.58	C 20:4 – Arachionic acid	1.5 ± 0.12	1.4 ± 0.02

*Note:* Results are mean ± standard error. Superscripts with different alphabets in each row signify statistical differences (*p* < 0.05).

Live feed significantly influences the fatty acid profiles of fish throughout different life stages [59]. DHA and the DHA/EPA ratio play a major role in enhancing the fish larval growth and survival [60]. In *A. cmfri* adults, EPA and DHA were present at 2.1 g and 3.8 g/100 g, respectively, yielding a DHA:EPA ratio of 1.81:1, close to ~2:1 recommended for most marine finfish larvae [61, 62]. In unenriched *Artemia* nauplii, their values were a meager 0.4 g and 0.1 g/100 g, respectively. Unenriched *Artemia* is typically known to contain very low DHA and modest EPA; consequently, its baseline DHA:EPA is far below 1 and requires enrichment for larval requirements [57]. As *A. cmfri* outperforms unenriched *Artemia* by naturally providing both EPA and DHA; therefore, reliance on enrichment logistics that often suffer from batch variability and oxidation losses [58] is expected to be reduced by using this copepod in the future.

Saturated fatty acids (SFAs), mono unsaturated fatty acids (MUFAs), and poly unsaturated fatty acids (PUFAs) constituted 39.2%, 31.6%, and 29.2% of the total fatty acids in *A. cmfri* adults and 26.7%, 37.9%, and 35.4% in unenriched *Artemia* nauplii. In PUFA, the contribution of ῳ3 and ῳ6 in *A. cmfri* adults and unenriched *Artemia* nauplii was 13.4 g and 15.8 g/100 g and 24.2 g and 11.2 g/100 g, with *n*‐3:*n*‐6 ratios of 0.85 and 2.16, respectively. Tropical paracalanoids carry relatively lower total lipid under culture (11–26 mg g^-1^) yet exhibit very favorable HUFA ratios, meeting larval recommendations [63]. Among the most studied tropical copepods, *P. crassirostris* exhibited the highest levels of EPA (2.8%) and DHA (6.1%), with Σ*n*‐3 of 10.7 g/100 g and a DHA:EPA ratio of 2.2:1. For *A. cmfri*, the total *n*‐3 PUFA (Σ*n*‐3 = 13.4 g/100 g) content was higher, but the *n*‐3/*n*‐6 ratio was lower due to the higher content of linoleic acid. *A. cmfri* also carries high palmitic acid (19.7%), above the typical copepod range (11%–17%) [14] and higher oleic acid (24.7%).

Amino acids, particularly EAAs, cannot be synthesized in sufficent quantities to meet the physiological requirements and hence need to be supplied through the diet [64]. Similarly, the fatty acid composition of larvae also depends on the live food and the fish’s ability to digest and absorb these nutrients. Their differences in feed cause a change in the amino acid and fatty acid composition of the larvae [65]. In turbot and sturgeon, larvae fed *Artemia* alone were reported to be majorly deficient in threonine, leucine, and phenylalanine [8, 66].

## 4. Conclusion

Collectively, these findings demonstrated that *A. cmfri* has a strong potential to be an alternative live feed for the larval rearing of marine finfishes, including rabbitfish, during the later phases of their rearing. Further research is required to optimize its use as a feed, including evaluating the effects of this novel prey on larval quality across diverse marine finfishes, developing methods for mass‐scale production, and exploring nutritional enhancement opportunities through appropriate enrichment protocols to establish standardized practices for aquaculture applications.

## Author Contributions

**Suresh Babu Padinhate Purayil:** conceptualization, methodology, writing – original draft preparation. **Mini Kalapurakkal Gopalan**: data curation. **Sanal Ebeneezar**, **Anuraj Anirudhan**, **Tanveer Hussain**, **Mahendra Pal, and Kiran Thomas Mathew**: investigation. **Sonali Suresh Mhaddolkar**, **Kalidas Chellappa, and Grinson George Padinjakara**: resources. **Suresh Vettath Raghavan**: writing – reviewing and editing. **Shubhadeep Ghosh**: supervision.

## Funding

The authors received no specific funding for this work.

## Conflicts of Interest

The authors declare no conflicts of interest.

## Data Availability

The data will be made available upon request.
